# Responsible conduct of research

**DOI:** 10.4314/ahs.v18i1.25

**Published:** 2018-03

**Authors:** Beuy Joob, Viroj Wiwanitkit

**Affiliations:** 1 Sanitation 1 Medical Academic Center, Bangkok Thailand; 2 Visiting professor, Hainan Medical University, China

Dear Editor, we read the publication on “responsible conduct of research” with great interest^1^. We would like to share ideas and experience from our country on this issue. First, the misconduct is not uncommon and can be seen worldwide. The role of an institute for management of the problem is very important since education seems to be the best way for prevention of the problem^2^. Nevertheless, the misconduct is sometimes performed by the seniors and it is usually hard to manage. In the community with rooted culture of junior-senior system, deep rooted culture becomes the big obstacle for ethical management of the misconduct^3^. No action and no responsibility to the problem are not uncommon. How to manage this important problem seen in many developing countries is a big challenge. Social network to watch the problem as the surveillance system, sharing information on unwanted misconduct episodes and collaborative sanctioning to the unethical persoms might be a new solution in the present era.
